# A Case Report of Monkeypox in a 4-Year-Old Boy from the DR Congo: Challenges of Diagnosis and Management

**DOI:** 10.1155/2020/8572596

**Published:** 2020-04-09

**Authors:** Anna Korsgaard Eltvedt, Michael Christiansen, Anja Poulsen

**Affiliations:** ^1^Global Health Unit, Department of Paediatric and Adolescent Medicine, Copenhagen University Hospital, Rigshospitalet, Copenhagen Ø, Denmark; ^2^Department of Congenital Disordes, Statens Serum Institut, Copenhagen S, Denmark

## Abstract

Monkeypox (MP) is a rare zoonotic disease that most commonly transmits from bush animals to humans in the Congo Basin of Africa. However, an increase in cases of MP has been observed over the past decades with frequent outbreaks as well as export of the disease out of the African continent. MP belongs to the same genus of viruses as smallpox, the *Orthopoxvirus*, and vaccination against smallpox gives some protection against MP. With the eradication of smallpox in 1980, vaccination against smallpox has ceased. The resulting decrease of immunity against *Orthopoxvirus* is thought to be related to the increase in MP cases. Furthermore, closer contact between humans and bush animals could play a role along with the ongoing difficulties of controlling HIV in the same geographical area. MP remains a diagnostic challenge. Lack of knowledge about the disease among health personnel plays an important role, as well as access to diagnostic tools is limited. Treatment of MP is for now symptomatic. We report the case of a 4-year-old boy from the DR Congo with the clinical diagnosis of MP. This case illustrates some of the abovementioned challenges related to the management of MP in the field.

## 1. Introduction

Monkeypox is a zoonotic disease occurring in Central and West Africa, especially in the DR Congo, where it is considered endemic. MP is most often transmitted to humans via bush animals (e.g., rodents) by direct contact between humans and animals. Interhuman transmission, previously regarded less important, may be on the rise [1].

MP belongs to the same viral genus as smallpox, and the clinical presentation is similar; however, MP is often less severe. Disease severity depends on the patient's comorbidities and age, and case fatality in MP has been reported up to 15%, with younger children being at highest risk [1, 2].

Also, the majority of HIV-positive patients living in MP endemic areas are either not aware of their status or not on sufficient treatment to suppress their viral load [3]. Immunocompromised individuals are more susceptible to MP [1]; hence, HIV may also be a contributing factor to the current rise in MP.

In the last decades MP has been on the rise with most recent outbreaks occurring in Nigeria (89 cases in 2017), the Republic of the Congo (88 cases in 2017), and Cameroon (16 cases in 2018) [4–6]. The DR Congo has reported >1000 cases annually since 2005 [4]. Also, in 2013, a local district in the DRC saw an increase of 600% in cases of human MP [7].

Human MP has been confirmed in individuals out of the African continent on several occasions too, in the USA in 2003 [8], in the UK in 2018 [9], in Israel in 2018 [10], and in Singapore in 2019 [11]. These cases were all imported from West Africa.

MP is challenging to manage in the field due to limited knowledge of the disease among patients and health staff and lack of diagnostic tools and treatment protocols. This not only limits the level of care offered to the affected individuals but may also put other patients and health personnel at risk of infection.

Based on the rise of reported cases of MP globally and the challenges related to diagnosis and management of MP in the field, we wish to bring this case forward as to keep raising attention to the need for a coordinated health approach in order to control this trend. This case illustrates the core of the challenges of the hands-on management of MP in the field.

## 2. Case Presentation

We report the case of a 4-year-old boy who was admitted to a referral hospital in the Northern DR Congo in December 2016.

Having been sick for 3 days, on admission day 1, he presented with low-grade fever (tp 37.9°C), rhinitis, conjunctivitis, cough, severe left-sided cervical lymphadenitis, and a non-itchy vesiculopapular rash with elements sized between 5 and 10 mm in diameter spread over his truncal area and face. He was alert and had a normal heart rate of 115 bpm and respiratory rate of 30/min. He was previously healthy, and he had received no vaccinations. He was not tested for HIV due to unavailability of treatment; hence, testing was considered unethical and discouraged by the medical leader of the project. Three adults in his village had similar but milder symptoms.

The child was first admitted to the general paediatric ward, but on day, 2 he was admitted to the isolation unit with his father on suspicion of chickenpox or measles. He was started on i.v. amoxicillin-clavulanic acid, retinol tablets, and antibiotic eye drops according to the local measles protocol. He also received paracetamol, diluted plumpynut, and i.v. maintenance fluids.

Over the next 2–5 days, he experienced fevers up to 38.5°C, and the rash worsened as all elements grew simultaneously penetrating 3–4 mm into his skin. The rash spread to cover his entire body surface, including palms, foot soles, and mucous membranes, the latter resulting in severe and painful stomatitis. He was changed to i.v. ceftriaxone, and pain management was intensified to include morphine.

In spite of supportive treatment, his condition gradually worsened with the skin and oral manifestations being his major problem, and the child passed away on admission day 12.

Means of blood analysis to detect measles, chickenpox, or monkeypox were not available at the local health facility. However, on admission day 2, a blood sample was sent to a diagnostic unit in the capital city of Kinshasa for examination of IgM and IgG against measles. Ten days later, the results came out negative for measles.

**Figure 1:**
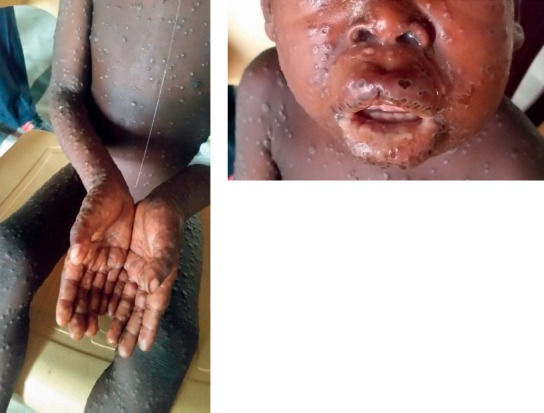
Photos of rash at admission day 5 (day 8 after onset of symptoms).

The local health personnel recognized this as a case of MP. This was supported by experts in paediatric infectious diseases in Denmark, who received photos and a written file by e-mail. However, in spite of clinical suspicion of MP, no other samples were sent for diagnostics. This was due to logistic challenges as well as fear among healthcare workers of handling contagious specimens and because of reluctancy from the project leader to carry out any more tests (see Figure 1).

Two weeks later, a 10-year-old boy from another village was seen at the same health facility with similar but milder symptoms. He was dismissed healthy one week later without any laboratory tests for MP done. An outreach team tried to track down a possible index person or other affected people in each village from where the children originated without result.

## 3. Discussion

MP is a disease resembling smallpox, and even if it is generally less severe, MP may still be accompanied by a number of complications ranging from skin and eye complications and pneumonia to systemic illness and death [2].

The management of MP represents challenges on both a local and a global level.

The reported case illustrates several of the obstacles related to the local management of the disease. This includes lack of recognition of the disease by local and expatriate health personnel and lack of diagnostic tools at rural health facilities (however, PCR on a blood sample performed in a centralized laboratory could have confirmed the case positive). Furthermore, the diagnostic process may be delayed by logistic challenges, i.e., lack of knowledge on how to collect and send a sample safely, as well as fear among staff of handling contagious specimens, which has also been reported in other countries [12]. Finally, cases may remain undiagnosed due to reluctancy among local and expatriate health leaders to put efforts into making a correct diagnosis, as the importance of this is not recognized. Hence, international surveillance data may be constrained.

In order to improve local management of MP, guidelines and raised awareness of the disease could help staff in carrying out correct management of patients suspected of MP. Besides improved supportive treatment, antiviral drugs such as tecovirimat may be a possible future treatment for MP [13].

On a global level, the rise of MP is worrisome. The most important reason for the rise in MP is thought to be the waning immunity to poxvirus following the cease of smallpox vaccination after 1980 [1, 2, 4]. Because the smallpox vaccine is expected to protect 85% against MP and the poxvirus vaccination has stopped, current exposed populations are less or not protected against MP [14]. Adding to this, HIV coinfection could also contribute to the rise in MP, as *Orthopoxvirus* is expected to have a more severe outcome in immunocompromised patients, e.g., in HIV-positive individuals [1, 15].

Even though West and Central Africa have a lower prevalence of HIV (1.5%) compared to the rest of sub-Saharan Africa, only 64% of people living with HIV in West and Central Africa are aware of their status, and nearly ¾th of the children living with HIV in this region do not have access to antiretroviral therapy [3] making children a high-risk population for MP and HIV coinfection.

Another factor contributing to the flare in MP may be due to closer contact between displaced human populations and bush animals caused by conflicts in MP-affected areas, climate change, and deforestation [4, 16].

The challenges of global surveillance of MP are also important to address. Due to diagnostic shortcomings and lack of reporting of cases to relevant health authorities, the current numbers could be underestimated.

Conclusively, the circumstances for MP to spread to humans seem to be enhanced calling for a coordinated cross-sectoral approach involving local and international health institutes.

With this case, we also wish to raise awareness about the disease among health personnel working in affected areas in order to keep MP as a differential diagnosis when consulting patients with an undefined rash.
